# P2Y_2_ receptor deficiency aggravates chronic kidney disease progression

**DOI:** 10.3389/fphys.2013.00234

**Published:** 2013-09-19

**Authors:** Sebastian A. Potthoff, Johannes Stegbauer, Jan Becker, P. Johannes Wagenhaeuser, Blanka Duvnjak, Lars C. Rump, Oliver Vonend

**Affiliations:** ^1^Department of Nephrology, Medical Faculty, University DuesseldorfDuesseldorf, Germany; ^2^Department of Pathology, Medizinische Hochschule HannoverHannover, Germany; ^3^Department of Radiology, Berufsgenossenschaftliches Universitätsklinikum BergmannsheilBochum, Germany

**Keywords:** purinercic receptors, P2Y_2_ receptor, subtotal nephrectomy, chronic kidney disease, adenosine triphosphate (ATP)

## Abstract

Purinergic signaling is involved in a variety of physiological states. P2 receptors are mainly activated by adenosine triphosphate (ATP). Activation of specific P2Y receptor subtypes might influence progression of kidney disease. To investigate the *in vivo* effect of a particular P2 receptor subtype on chronic kidney disease progression, subtotal nephrectomy was performed on wild type (WT) and P2Y_2_ receptor knockout (KO) mice. During the observational period of 56 ± 2 days, survival of KO mice was inferior compared to WT mice after SNX. Subtotal nephrectomy reduced creatinine clearance in both groups of mice, but the decrease was significantly more pronounced in KO compared to WT mice (53.9 ± 7.7 vs. 84.3 ± 8.7μl/min at day 56). The KO mice also sustained a greater increase in systolic blood pressure after SNX compared to WT mice (177 ± 2 vs. 156 ± 7 mmHg) and a 2.5-fold increase in albuminuria compared to WT. In addition, WT kidneys showed a significant increase in remnant kidney mass 56 days after SNX, but significant attenuation of hypertrophy in KO mice was observed. In line with the observed hypertrophy in WT SNX mice, a significant dose-dependent increase in DNA synthesis, a marker of proliferation, was present in cultured WT glomerular epithelial cells upon ATP stimulation. Markers for tissue damage (TGF-β 1, PAI-1) and proinflammatory target genes (MCP1) were significantly upregulated in KO mice after SNX compared to WT SNX mice. In summary, deletion of the P2Y_2_ receptor leads to greater renal injury after SNX compared to WT mice. Higher systolic blood pressure and inability of compensatory hypertrophy in KO mice are likely causes for the accelerated progression of chronic kidney disease.

## Introduction

Nucleotides, predominantly adenosine triphosphate (ATP) and uridine triphosphate (UTP), are the ligands for P2 receptors. Intracellular ATP serves as the main source of energy and is crucial for almost all cell functions. Released from either neurons as neurotransmitters or in a paracrine fashion from a variety of cells and tissues, these nucleotides activate P2 receptors to trigger intracellular pathways (Ralevic and Burnstock, 1991; Burnstock, 1995; Oberhauser et al., 1999; Vonend et al., 2002; Ralevic and Burnstock, 2003; Vonend et al., 2003; Burnstock, 2006).

In almost every organsystem expression of several P2 receptor subtypes can be found (Turner et al., 2003; Burnstock and Knight, 2004). Activation of P2 receptors plays a role in transepithelial transport, proliferation, migration, platelet aggregation and has a potential influence on cardiovascular diseases. There are ligand-gated ion channel P2X receptors (P2X_1−7_) and G-protein coupled P2Y receptors (P2Y_1, 2, 4, 6, 11−14_) (Abbracchio et al., 2006; Burnstock, 2012). In the kidney in particular, the expression of multiple P2 receptors subtypes can be observed (Bailey et al., 2000; Vonend et al., 2003, 2005).

P2 receptors play a pivotal role within the sympathetic nervous system (Vonend et al., 2005; Gourine et al., 2009). As ATP serves as a co-transmitter from sympathetic nerve endings, activation of these P2 receptors occurs in sympathetic overactivity. It is well-known that patients with chronic kidney disease have an increased sympathetic activity (Rump et al., 2000; Vonend et al., 2002). In addition to ATP release from sympathetic nerve endings, endothelial dysfunction and increased shear stress contribute to a higher extracellular abundance of ATP, released from the endothelium in chronic kidney disease (Wan et al., 2008; Yamamoto et al., 2011).

There is evidence that P2Y receptor subtypes are important for maintaining renal function. In particular the P2Y_2_ receptor is involved in glomerular cell proliferation and modulation of renal tubule function including natriuresis (Harada et al., 2000; Vonend et al., 2002; O'Mullane et al., 2009; Booth et al., 2012).

Due to the role of P2Y_2_ receptors in vascular and tubule function, inflammation and renal cell proliferation, we hypothesize that activation of P2Y_2_ receptors plays an important role in the pathophysiology and the progression of chronic kidney disease.

The model of subtotal nephrectomy (SNX) is characterized by increased blood pressure and sympathetic overactivity (Potthoff et al., 2008; Hoch et al., 2011). In this study, for the first time, using this model in P2Y_2_ receptor knockout mice (P2Y_2_-R KO), we assessed the effects of P2Y_2_ receptor on the progression of chronic kidney disease.

## Methods

### Animal care

The P2Y_2_-R KO mouse was generously provided by Dr B. H. Koller (University of North Carolina, Chapel Hill, USA). Mice were generated by homologous recombination in embryonic stem cell lines. Northern blot RNA analysis from kidney lysates confirmed the complete loss of P2Y_2_-R expression in these mice (Homolya et al., 1999). The mice were originally on a B6D2 genetic background and back crossed with a SV129 mouse strain for 10 generations before starting the study. Mice homozygous for P2Y_2_-R KO were used in this study. Genotyping was performed in each individual mouse confirming the presence of the disrupted P2Y_2_-R gene (Homolya et al., 1999). Wild type (WT) littermates were used as controls. At the date of surgery, all mice were 62 ± 3 days old and weighed 21–26 g. Only male mice were used in this study. The investigations and surgery were performed in accordance with institutional guidelines. The animals were housed in type III Makrolon polycarbonate cages at 45% humidity, 20–22°C temperature and a 12 h day-night-cycle with free access to water and food. Standard food was *Altromin* (*Altromin 1314*: 20% protein, 0.4% NaCl, Lage/Lippe, Germany).

### Subtotal nephrectomy and sham surgery

Sham surgery or subtotal nephrectomy (SNX) was performed at 62 ± 3 days of age. WT mice and P2Y_2_-R KO mice were randomly allocated to either sham surgery or SNX. A Kaplan-Meier survival curve was generated from the survival data in the sham-surgery and SNX groups.

The mice were anesthetized with ketamin and xylazine by intraperitoneal injection (0.168 mg/g and 8 mg/g bodyweight, respectively). Subtotal nephrectomy was performed as previously described (Hoch et al., 2011). In brief, both kidneys were exposed by dorsal incision. The right kidney was removed completely. Two-thirds of the functional tissue from the left kidney was removed by ligation of the upper and lower renal poles leading to a subtotal reduction of functional renal tissue. Sham surgery was performed only by skin and abdominal muscle incisions.

### Preparation of urinary, serum and tissue samples

The mice were sacrificed 56 ± 2 days after surgery. Blood samples were gathered by retro orbital vein puncture on day 28 ± 2 and on the day of sacrifice. Serum samples were obtained from centrifuged blood samples. Twenty-four hour-urine was collected on day 0, 28 ± 2 and on the day of sacrifice using metabolic cages (*Techniplast*, Italy) for urinary albumin, creatinine and urea measurement.

On day 56 ± 2, mice were sacrificed, all organs were perfused with ice-cold PBS from the left ventricle and SNX-kidneys and sham-surgery kidneys were removed and embedded in paraffin. Tissue samples for RNA expression analysis were removed from the kidneys prior paraffin embedding.

### Systolic blood pressure measurement

Systolic blood pressure (BP) was measured non-invasively by tail-cuff sphygmomanometer using a *BP-98A* device (*Softron*, Japan). Mice were trained for 4 days prior to evaluation of BP. BP was measured on day 0, 28 ± 2 and 56 ± 2 prior to sacrifice.

### Kidney hypertrophy

Kidney weight was evaluated on the day of surgery (SNX groups) and at the end of study. The remnant kidney weight directly after SNX was estimated by calculating the kidney weight of right kidney minus the weight of the removed tissue from the left kidney. Change in kidney weight from the day of surgery (SNX) until sacrifice was regarded as an index of kidney hypertrophy.

### Quantification of serum and urine albumin and urea

Measurement of creatinine levels in serum and urine was performed using an enzymatic standard test supplied by *Labor&Technik*, Germany. The analysis was performed following the manufacturer's protocol. Serum and urinary urea and serum albumin were measured using standard tests supplied by *Randox*, Germany. The methods were followed according to the manufacturer's protocol. Urinary albumin levels were measured using the *Albuwell M Kit* (*Exocell*, USA). Urine albumin-to-creatinine ratio (UACR) was calculated for all urine samples (24-h urine collection samples using metabolic cages). Creatinine-clearance was calculated from urine volume, urinary- and serum-creatinine values.

### Proliferation assay

[^3^H]-thymidine assay was used to estimate proliferative activity in cells (Vonend et al., 2003). WT human glomerular epithelial cells were obtained from *Cambrex Corporation*, USA. For proliferation assays, cell passages ranging from 5 to 12 were used. Cells were housed at 37°C and 5% CO_2_. Cells were distributed on a 96-well-plate (150.000 cells/well) and grown for 3 days in cell culture medium containing 5% fetal calf serum until 70–80% confluence. At day 3, cells were starved for 24 h by reducing fetal calf serum content to 0.5%. ATP was added to resting cells (4 wells for each concentration) for 24 h (0–100 μM).

The cells were incubated with [^3^H]-thymidine (1 μCi/ml) during the last 6 h. Cells were washed three times with phosphate buffered saline, twice with ice-cold 10% trichloroacetic acid, and the fixed cellular material was solubilized in 1 ml 0.5 m NaOH for 2 h and mixed with 4 ml scintillation fluid (*Ultima-Gold, Canberra Packard*, Frankfurt, Germany) to measure the amount of radioactivity. Data are expressed as the mean ratio of radioactivity present in the 4 wells with identical concentration divided by control (0 μM ATP) values (% of control).

### RNA extraction and reverse transcription polymerase chain reaction

WT human glomerular epithelial cells were homogenized and total RNA was isolated with *Trizol-Reagent* (*Invitrogen*, Germany). Following DNA digestion (*Rnase free DNase/Invitrogen*), mRNA was isolated from total RNA with the *PolyATract* mRNA isolation system (*Promega*). cDNA was synthesized according to suppliers' protocol using oligo-dt primer and the reverse transcriptase superscript (*Invitrogen*). The amplification was performed with specific primers for P2Y_1, 2, 4, 6, 11_ receptor subtypes with 10% of the first strand cDNA and 1.5 units of *Platinum Taq-Polymerase* (*Invitrogen*) in a volume of 50 μl. After 5 min at 95°C followed by 35 cycles consisting of 1 min at 95°C, 1 min at 52–58°C, 1 min at 72°C and for termination 8 min at 72°C, 10 μl of the reaction products were analyzed on a 1.5% agarose gel containing ethidium bromide.

#### Quantitative real time PCR (qPCR)

SNX kidney samples were used to analyze relative expression levels for transforming growth factor beta 1 (TGF-β 1), plasminogen activator inhibitor 1 (PAI-1), nuclear factor kappa-light-chain-enhancer of activated B-cells (NFκ B), monocyte chemoattractant protein 1 (MCP1), cyclooxygenase 1 (COX1) and prostaglandin E synthase 1 (PGES1).

After homogenization of tissue with a *Tissue Ruptor* (*Qiagen*, Germany), total RNA was isolated using a *RNA Micro Kit* (*Qiagen*, Germany) according to the manufacturer's instructions. Quantitative real time PCR was performed with an *ABI PRISM 7300* (*Applied Biosystem*, Germany) and the *SYBR Green master mix* (*Qiagen*, Germany). The PCR reaction was performed in a total volume of 20 μl with 1 μl cDNA corresponding to 50 ng RNA as template.

The two-step PCR conditions were 2 min at 50°C, 15 min at 95°C, followed by 40 cycles (denaturation at 94°C for 15 s; annealing at 55°C for 30 s and extension at 72°C for 34 s). Experiments were performed in triplicate. 18S ribosomal RNA was chosen as the endogenous control (housekeeping gene). The levels of targeted genes were normalized to 18S rRNA expression and analyzed by REST 2008 V2.0.7 software.

The following *Taqman assays* were used: 18S (Mm03928990_g1), PAI-1 (Mm00435860_m1), TGF-β 1 (Mm01178820_m1), NFκ B (Mm01297400_m1), MCP1 (Mm00441242_m1), COX1 (Mm0047214_m1), PGES1 (Mm00452105_m1).

#### Statistical analysis

All data are expressed as means ± standard error of mean (SEM). *n* applies to the number of mice/tissue samples used in each group. Datasets were analyzed using SPSS 19.0 software. Multiple comparison of more than two groups was performed by One-Way ANOVA followed by Bonferroni's multiple comparison *post-hoc* test, where applicable. Values of *p* < 0.05 were considered as significant. If applicable, a higher level of statistical significance is stated (*p* < 0.01, *p* < 0.001).

qPCR data was statistically analyzed using REST 2008 V2.0.7 software.

Comparison of survival was calculated using the logrank test (Mantel-Cox test).

## Results

### Survival

Renal function in the model of subtotal nephrectomy is the major contributing factor determining animal survival. To evaluate a general role of P2Y_2_ receptor in the progression of chronic kidney disease, we registered the survival of sham surgery and SNX mice.

**Figure 1:**
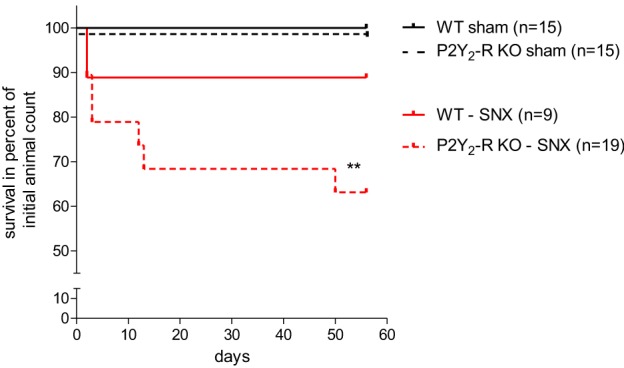
**Kaplan-Meyer-survival curve of all groups: sham surgery groups showed 100% survival after 56 ± 2 days.** In the SNX surgery groups, more WT mice survived during the observation period than P2Y_2_-R KO mice (88.9 vs. 63.1%). Comparing this data to the sham surgery groups shows that subtotal nephrectomized P2Y_2_-R KO mice had a significantly reduced survival (^**^p < 0.01) whereas subtotal nephrectomized WT mice showed no significantly reduced survival compared to sham surgery WT mice (*p* = NS).

Observational period for all mice was 56 ± 2 days after surgery. Overall survival was 100% in both sham surgery groups (Figure 1). During the observation period, 1 out of 9 WT mice died after SNX compared to 7 out of 19 in the P2Y_2_-R KO group (survival rate WT vs. P2Y_2_-R KO: 88.9 vs. 63.1%) (Figure 1). Comparing the survival with the WT sham surgery group, survival was not significantly different in WT mice (survival rate 88.9 vs. 100%, *p* = NS). However, survival was significantly reduced in P2Y_2_-R KO mice which underwent SNX compared to KO sham surgery mice (survival rate 63.1 vs. 100%, *p* < 0.01, X^2^ = 6.658, *df* = 1).

### Systolic blood pressure

Increase in blood pressure occurs in chronic kidney disease. It correlates well with the stage of chronic kidney disease. A high blood pressure also contributes to the progression of kidney disease.

**Figure 2:**
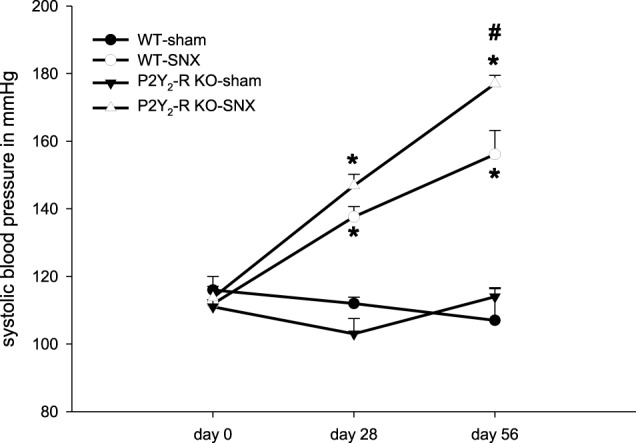
**Systolic blood pressure on day 0 (before surgery), day 28 and day 56 for each group: Sham surgery groups did not show any significant change in systolic blood pressure.** SNX groups showed a significant rise in systolic blood pressure on day 28 and 56 compared to day 0 (^*^p < 0.05). At day 56, systolic blood pressure was significantly higher in P2Y_2_-R KO SNX compared to WT SNX mice (^#^*p* < 0.05) (mean ± SEM).

Systolic blood pressure was measured at three different time points (day 0, 28 ± 2, 56 ± 2) using an automated tail-cuff sphygmomanometer. At baseline before surgery (day 0), all four groups showed no difference in systolic blood pressure. Neither sham surgery groups showed a rise in systolic blood pressure during the observation period. In contrast, the SNX groups had a significant elevation of systolic blood pressure (day 28 and 56). In addition, systolic blood pressure was significantly higher in P2Y_2_-R KO mice after SNX compared to WT SNX mice (day 56, WT vs. P2Y_2_-R KO: 156 ± 7 vs. 177 ± 2 mmHg) (Figure 2).

### Renal function

In order to evaluate renal function, measurement of renal retention parameters and endogenous creatinine clearance was performed in all groups.

**Figure 3:**
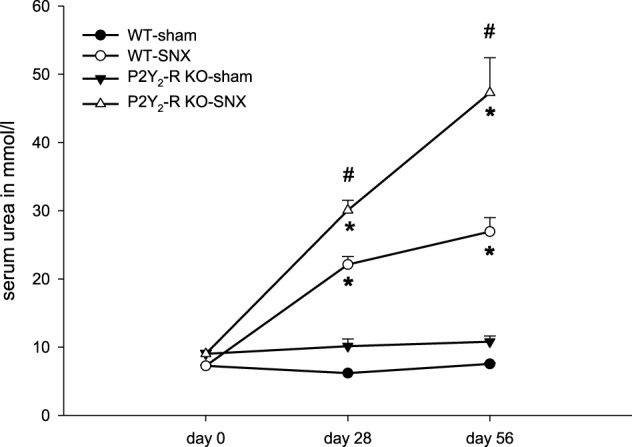
**Serum urea on day 0 (before surgery), day 28 and day 56 for each group: sham surgery groups did not show any significant change in serum urea levels.** SNX groups showed a significant rise in serum urea levels on day 28 and 56 compared to day 0 (^*^p < 0.05). At day 28 and 56, serum urea levels were significantly higher in P2Y_2_-R KO SNX compared to WT SNX mice (^#^*p* < 0.05) (mean ± SEM).

During the observation period (day 28 ± 2, 56 ± 2) there was a significant increase in serum urea in both SNX groups compared to baseline (day 0). There was no significant change of serum urea in sham surgery groups (Figure 3). At day 56 ± 2, serum urea was 3.6-fold higher in WT SNX compared to WT sham surgery mice and 4.4-fold higher in P2Y_2_-R KO SNX compared to KO sham surgery mice. Accordingly, serum urea was significantly higher in P2Y_2_-R KO SNX mice compared to WT SNX mice. This is in line with the observed creatinine clearance at day 56: creatinine clearance was significantly higher in WT SNX mice compared to P2Y_2_-R KO SNX mice.

All assessed physiological parameters are summarized in Table 1.

**Table 1:** **Serum and urine parameters from all groups after 56 ± 2 days**.

**Parameter**	**Units (SI)**	**WT-SNX**	**P2Y_2_–R KO-SNX**	**WT-sham**	**P2Y_2_-R KO-sham**
Serum creatinine	μmol/l	19.5 ± 3.5	24.8 ± 2.7	9.7 ± 0.7	4.4 ± 0.4
Serum urea^*^	mmol/l	27.1 ± 2.1	47.4 ± 5.2	7.5 ± 0.1	10.8 ± 0.8
Urine volume (24 h)	ml	1.8 ± 0.2	2.6 ± 0.4	0.4 ± 0.1	0.2 ± 0.1
Creatinine clearance^*^	μl/min	84 ± 9	54 ± 8	114 ± 14	112 ± 29
Urine albumin-to-creatinine ratio^*^	mg/mg	2.0 ± 0.5	5.1 ± 0.8	0.02 ± 0.00	0.05 ± 0.02

Parameters marked with

“*” *show a significant difference (p < 0.05) comparing WT and P2Y_2_-R KO SNX groups (mean ± SEM)*.

### Albuminuria

Mice that underwent subtotal nephrectomy are characterized by the progression of chronic kidney disease. The histologic equivalent in tissue injury is the onset and progression of glomerulosclerosis and is accompanied by albuminuria.

**Figure 4:**
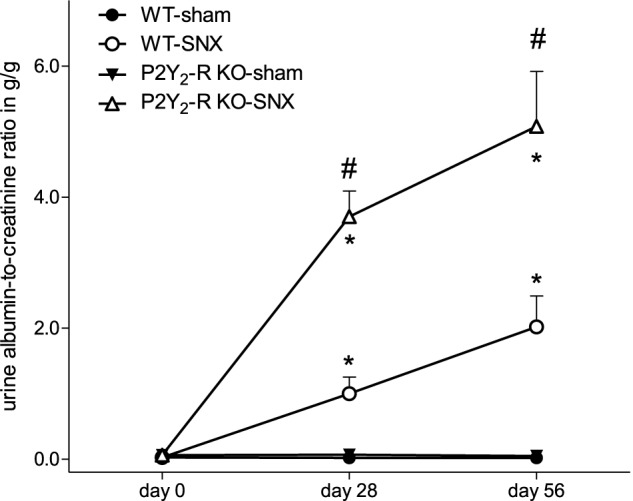
During the observation period, UACR significantly increased in the SNX groups compared to the sham surgery groups (day 28 and 56, ^*^p < 0.05). In addition, UACR was significantly higher in P2Y_2_-R KO SNX compared to WT SNX (^#^*p* < 0.05) (mean ± SEM).

Albuminuria, estimated by UACR, was almost absent in sham surgery groups at all time points. In contrast, compared to baseline, there was a significant rise in albuminuria in the SNX groups (Figure 4). In addition, the rise in UACR was more pronounced in P2Y_2_-R KO SNX mice. After 56 days, the UACR was more than 2.5-fold higher in KO SNX compared to WT SNX mice (Table 1, Figure 4).

Marked differences in glomerulosclerosis between P2Y_2_-R KO SNX and WT SNX mice could not be detected (data not shown).

### Gene expression

Kidney cortex tissue from SNX mice at day 56 was assessed for the expression of specific genes which are associated with inflammatory response and which are related to the function of P2Y_2_-receptor in the immune system. In addition, gene expression was assessed for genes related to kidney injury.

**Figure 5:**
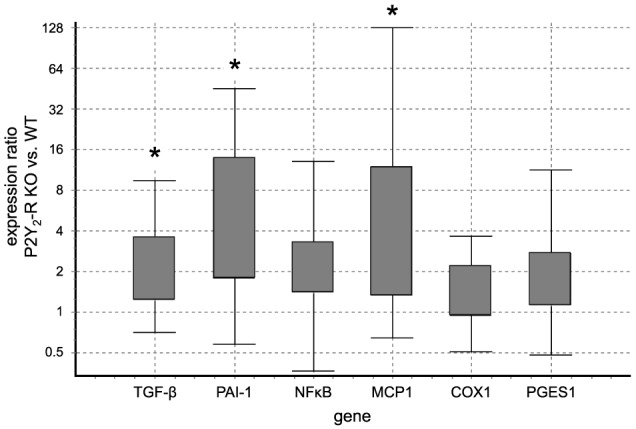
**Gene expression ratio of target genes indicating tissue damage and inflammation in WT SNX vs. P2Y_2_-R KO SNX mice assessed by qPCR: positive values indicate fold-change in P2Y_2_-R KO SNX over WT SNX expression levels.** TGF-β 1, PAI-1 and MCP1 showed a significantly higher expression in P2Y_2_-R KO SNX than WT SNX mice (^*^p < 0.05). (Boxes represent the interquartile range. Whiskers represent the minimum and maximum observations. Analysis was performed with REST 2008 V2.0.7 software).

In qPCR analysis, wecompared the expression level of specific target genes in P2Y_2_-R KO SNX mice to the expression in WT SNX mice. qPCR results were normalized to 18S ribosomal RNA expression. Comparison revealed a significant increase in expression ratio for TGF-β 1, PAI-1 and MCP1 in P2Y_2_-R KO SNX mice (Table 2, Figure 5).

**Table 2:** **Relative gene expression normalized to 18S expression levels comparing WT and P2Y_2_-R KO SNX mice**.

**Gene**	**Expression**	***SE***	***p*-value**	**Result**
18S	1.000			
TGF-β 1	2.191	0.999–4.524	0.047	^*^(UP)
PAI-1	4.332	1.369–22.287	0.010	^*^(UP)
NF-κ B	2.067	1.096–4.861	0.070	
MCP1	4.187	1.102–32.674	0.024	^*^(UP)
COX1	1.443	0.762–2.621	0.165	
PGSE1	1.778	0.858–3.297	0.122	

*Values higher than 1 indicate higher expression in P2Y_2_-R KO mice. Data analysis was performed using REST 2008 V2.0.7 software*.

* *p < >0.05*.

### Hypertrophy of kidney tissue

Loss of kidney tissue results in the hypertrophy of the healthy remnant kidney, partially compensating for the loss of functional tissue. Since the P2Y_2_-receptor is involved in cell proliferation, we assessed the increase in kidney weight.

In SNX mice, at day 0, total kidney weight (two kidneys) was reduced to a similar extent in WT and KO mice (WT: −69.3 ± 1.35%; P2Y_2_-R KO: −68.7 ± 1.1%).

**Figure 6:**
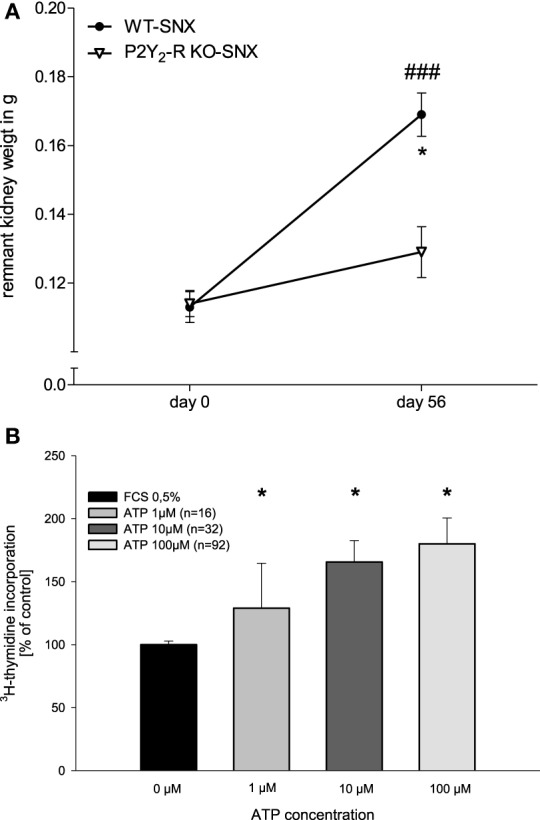
**(A)** Remnant kidney weight in SNX groups at day 0 and day 56 (^*^p < 0.05 compared to day 0; ^###^*p* < 0.001 WT SNX vs. P2Y_2_-R KO SNX). **(B)** [^3^H]-thymidine insertion in WT human glomerular epithelial cells in dependence of ATP stimulation (0–100 μM) (^*^p < 0.05 compared to 0 μM ATP).

At day 56, SNX kidney weight was measured and compared to the estimated weight at day 0. Comparison of the SNX groups showed a significant difference at day 56 (Figure 6A). Remnant kidney weight in WT SNX mice increased significantly during the observational period (day 56, 150 ± 6% compared to day 0, *p* < 0.05). In contrast, SNX in P2Y_2_-R KO mice led to a minor, non-significant increase in remnant kidney weight (day 56, 113 ± 6% compared to day 0, *p* = NS).

### P2Y receptor expression and proliferation assay in human glomerular epithelial cells

To illustrate the proliferative effect explaining the observed hypertrophy in the WT mice with functional P2Y_2_ receptor after SNX, we assessed P2Y-receptor status in glomerular epithelial cells and the measured insertion of [^3^H]-thymidine upon ATP stimulation in order to estimate DNA synthesis which is an indicator of cell proliferation. RT-PCR with specific primers for P2Y_1_, P2Y_2_, P2Y_4_, P2Y_6_, P2Y_11_ receptors confimed the expression of these receptor subtypes in WT human glomerular epithelial cells (data not shown).

Human glomerular epithelial cells were stimulated with increasing doses of ATP (1–100 μM) in an *ex vivo* assay. Compared to non-stimulated controls, ATP induced a significant, dose dependent increase in DNA synthesis up to 180.1 ± 20.5% (Figure 6B).

## Discussion

Chronic kidney disease is a major health burden and has a major impact on cardiovascular morbidity and mortality (Matsushita et al., 2010). Progression of kidney disease is vastly dependent on two factors: inflammatory response and fibrotic remodeling. The progression of chronic kidney disease is linked to a progressive scarring of glomeruli and subsequent development of tubulointerstitial fibrosis (Ma and Fogo, 2003). This is accompanied by albuminuria, an independent predictor for cardiovascular events (Matsushita et al., 2010). Diseased kidneys activate afferent sensory nerves, which project to the sympathetic nuclei in the central nervous system. This leads to increased sympathetic activity in organs like the heart, blood vessels but also kidneys (Rump et al., 2000). In chronic kidney disease, sympathetic overactivity, mechanical stress and inflammatory responses from various cell types within the kidney induce the release of extracellular ATP (Brecht et al., 1976; Gerasimovskaya et al., 2002; Oberg et al., 2004; Kotanko, 2006; Suliman and Stenvinkel, 2008; Masuo et al., 2010). This excess of extracellular ATP likely leads to activation of various P2 receptor subtypes. However, it remains unclear whether activation of P2 receptors modulates the outcome in chronic kidney disease. A P2 receptor subtype, which has been associated with nephron function and renal cell proliferation, is the P2Y_2_ receptor (Harada et al., 2000; Vonend et al., 2003; Pochynyuk et al., 2008). This study elucidates the P2Y_2_ receptor's role in a model of chronic kidney disease.

The decreased survival in P2Y_2_-R KO mice after SNX is one of the most striking findings, which indicates a general role of the P2Y_2_ receptor in chronic kidney disease. To evaluate the underlying cause of this difference, we assessed blood pressure, physiological parameters and histology throughout the study.

Chronic kidney disease is associated with increased blood pressure due to increased sympathetic activity and activation of the renin angiotensin system (Mailloux, 2001; Vonend et al., 2008; U.S. Renal Data System, 2011). It has been shown previously that mice develop hypertension after SNX (Ma and Fogo, 2003). In our study, sham surgery and SNX mice showed a similar systolic blood pressure at baseline. In sham surgery mice, systolic blood pressure remained stable during the observation period. In the SNX groups, blood pressure significantly increased over time. Interestingly, P2Y_2_-R KO mice developed a significantly higher blood pressure compared to WT SNX mice.

P2Y_2_ receptor activation is a crucial element in a variety of physiological mechanisms that influence blood pressure. These mechanisms include endothelium function and vasoconstriction in the vasculature, modulation of inflammatory response of immune cells and modulation of epithelial function. The P2Y_2_ receptor is expressed along the nephron and plays an important role in tubule epithelial function (Unwin et al., 2003). In the distal nephron, Ponchynyuk et al. showed that P2Y_2_ receptor activation decreases the open probability of the epithelial sodium channel (ENaC) leading to increased natriuresis and water excretion (Pochynyuk et al., 2008, 2010). Rieg et al. then demonstrated that P2Y_2_ receptor activation causes an immediate drop in blood pressure and increases the fractional sodium excretion. In P2Y_2_-R KO mice, sodium excretion was reduced compared to WT mice. This also caused a decrease in free water excretion. Rieg et al. could not observe any difference in glomerular filtration rate at baseline or upon stimulation of P2Y_2_ receptor, nor could they detect any difference in mean blood pressure at baseline comparing WT and KO mice (Rieg et al., 2011). In this study, Rieg et al. used P2Y_2_-R KO mice on a SV129 background, using mice from the same source as mice used in our study. The results are consistent with our findings, which show no significant difference in systolic blood pressure of P2Y_2_-R KO compared to WT mice at baseline. However, in an earlier study, using mice on a C57Bl/6 background, significantly higher baseline systolic blood pressure in these KO mice was observed (Rieg et al., 2007). Rieg et al. assumed that the genetic background might explain this difference. Even though basal systolic blood pressure did not differ in our study, in P2Y_2_-R KO SNX mice, systolic blood pressure was significantly higher at the end of the observational period compared to WT SNX mice. This might be explained by the lack of P2Y_2_ receptor activation and its effects on natriuresis and water excretion.

Another factor that might contribute to the elevated blood pressure in P2Y_2_-R KO mice is the impact of endothelial vasodilatation upon P2Y_2_ receptor activation. This response is driven by endothelium-derived hyperpolarizing factor and nitric oxide production (Marrelli, 2001; Buvinic et al., 2002; da Silva et al., 2009; Raqeeb et al., 2011). Impaired endothelial vasorelaxation is a hallmark of endothelial dysfunction which occurs in the setting of chronic kidney disease. Therefore, it is feasible that P2Y_2_ receptor deficiency in chronic kidney disease contributes to the impaired endothelial function resulting in elevated systolic blood pressure as observed in our study.

In our study, progression of kidney disease after SNX was more pronounced in P2Y_2_-R KO mice than in WT controls. Creatinine clearance was significantly reduced, and both albuminuria and serum urea were significantly higher in P2Y_2_-R KO mice after SNX. Since increased blood pressure accelerates the decline of kidney function and increases proteinuria, the difference in systolic blood pressure is likely to be one of the contributing factors for the observed outcome (Young et al., 2002; Matsushita et al., 2010).

However, if increased systolic blood pressure was the sole cause for worse outcome in the P2Y_2_ receptor KO group, a marked difference in glomerulosclerosis should be present in these mice. Histological assessment of the kidneys could not reveal any difference in glomerulosclerosis (data not shown). This finding suggests that mechanisms other than increased blood pressure contribute to the marked difference in renal function between P2Y_2_-R KO and WT SNX mice.

As stated above, progression of kidney disease is also dependent on the inflammatory response to kidney injury. It has been shown that P2Y_2_ receptors have a direct impact on inflammation and proliferation.

In order to delineate mechanisms other than increased systolic blood pressure, mRNA expression was analyzed at day 56 after SNX. Markers indicating increased maladaptive changes were significantly increased in P2Y_2_-R KO SNX mice. TGF-β 1 and PAI-1 showed a significant increase at day 56 after SNX in the P2Y_2_-R KO group (Figure 5). TGF-β 1 not only reflects increased tissue damage but can act as a strong inducer of inflammation (Lan and Chung, 2012).

The progressive decline in kidney function and the differences between WT and KO SNX mice in our study, might be associated with modulation of the inflammatory response (Suliman and Stenvinkel, 2008). P2Y_2_ receptors are among the P2 receptors that show markedly increased expression in macrophages after induction of inflammatory response indicating a potential role of the P2Y_2_ receptor in the regulation of inflammation (Luttikhuizen et al., 2004). Others showed that P2Y_2_ receptor activation increases the expression of cyclooxygenase-1/2 and release of prostaglandin E_2_ (PGE2) which inhibits the release of pro-inflammatory cytokines from macrophages and T-lymphocytes (Welch et al., 2003; Degagne et al., 2009; Kalinski, 2012). Furthermore, administration of the P2Y_2_ receptor agonist UTP greatly reduced the infiltration of neutrophils after myocardial infarction, an effect which was abolished in P2Y_2_-R KO mice (Cohen et al., 2011). Therefore, activation of P2Y_2_ receptors in an inflammatory setup can limit the extent of inflammatory response. Since a sustained inflammation contributes to the progression of chronic kidney disease, deficiency of P2Y_2_ receptors might be detrimental for kidney function.

In order to assess effects on inflammation, we analyzed mRNA expression of Nfκ B, MCP1, PGSE1, and COX1 in SNX kidney cortex samples. Nfκ B, PGSE1, and COX1 showed a statistically non-significant tendency toward higher expression in P2Y_2_-R KO SNX mice. It is well-known that in this model, differences in mRNA levels in late observational periods tend to be subtle, since a steady state with slow progression of kidney disease has been established (Rumberger et al., 2007). This might partially explain the non-significant differences in some of the inflammatory markers in qPCR assessment. Interestingly, at day 56, MCP1 expression was higher in P2Y_2_-R KO SNX compared to WT SNX mice, which indicates an increased inflammatory response in the KO SNX group. Since sustained inflammation accelerates the progression of chronic kidney disease, this might partially explain the worse outcome in P2X_2_-R KO mice after SNX.

In addition to modulating inflammation, P2Y receptors are also involved in the regulation of proliferation and hypertrophy. Vonend et al. showed that P2Y receptor activation promotes proliferation of human mesangial cells (Vonend et al., 2003). In our study, WT SNX mice showed a significant increase in remnant kidney weight whereas remnant kidneys in P2Y_2_-R KO SNX mice failed to show a significant increase in renal mass. These adaptive processes are sustained by a proliferation of different renal cell types, leading to an increase in glomerular size and increase in tubular tissue, which can partially compensate for the reduced GFR and therefore counteract the decline in kidney function (Waldherr and Gretz, 1988; Hoy et al., 2003; Alperovich et al., 2004). In the absence of the P2Y_2_ receptor, these adaptive mechanisms seem to be abolished and could partially explain the lower creatinine clearance in the KO SNX mice.

The balance between proliferation and apoptosis depends upon the concentration of extracellular ATP and on the subtype of stimulated P2-receptors (Harada et al., 2000). Extracellular ATP leads to a dose-dependent proliferation of mesangial cells in a manner that is P2Y_2_ receptor dependent (Harada et al., 2000). In our study, deficiency of P2Y_2_ receptor abolished most of the remnant kidney hypertrophy following SNX. In addition, albuminuria was significantly higher in the P2Y_2_-R KO group. To illustrate the proliferative effect explaining the observed hypertrophy in the WT mice with functional P2Y_2_ receptor after SNX, we observed a dose dependent increase in cell proliferation by ATP stimulation in glomerular epithelial cells. These cells are essential for the structure of the glomerular filtration barrier and their integrity is important to prevent albuminuria. The lack of compensatory hypertrophy might contribute to the worse outcome after subtotal nephrectomy in the P2Y_2_ receptor deficient group.

In summary, P2Y_2_ receptor deficiency is detrimental in chronic kidney disease. This is most likely caused by increased elevation of systolic blood pressure. The P2Y_2_ receptor also modulates inflammation and proliferation which may also contribute to the progression of chronic kidney disease in our model. We conclude that the P2Y_2_ receptor is crucial for blood pressure regulation and response to renal injury after induction of chronic kidney disease. Therefore, the P2Y_2_ receptor stimulation might be a potential therapeutic target.

### Conflict of interest statement

The authors declare that the research was conducted in the absence of any commercial or financial relationships that could be construed as a potential conflict of interest.

## Abbreviations

ATP: adenosine triphosphate
BP: blood pressure
DNA: deoxyribonucleic acid
FCS: fetal calf serum
h: hour
KO: knock out
NaOH: sodium hydroxide
NS: non-significant
PBS: phosphate buffered saline
PCR: polymerase chain reaction
SNX: subtotal nephrectomy
UTP: uridine triphosphate
WT: wild type.
